# Predictive study of machine learning combined with serum Neuregulin 4 levels for hyperthyroidism in type II diabetes mellitus

**DOI:** 10.3389/fonc.2025.1595553

**Published:** 2025-07-16

**Authors:** Huilan Gu, Ye Lu

**Affiliations:** Department of Endocrinology, Suzhou Ninth People’s Hospital, Suzhou, China

**Keywords:** SVM, CNN+LSTM model, Nrg4, T2DM complicated by FT, ultrasound images, classification

## Abstract

**Background:**

Neuregulin 4 (NRG4) is a novel metabolic regulator closely associated with insulin resistance and thyroid dysfunction. However, its role in the pathogenesis of comorbid type 2 diabetes mellitus and hyperthyroidism (T2DM-FT) remains to be systematically elucidated. Given the complex clinical characteristics of T2DM-FT patients, traditional statistical methods are often insufficient to effectively analyze nonlinear relationships among multiple variables. Machine learning techniques have garnered widespread attention due to their advantages in modeling high-dimensional, heterogeneous data.

**Objective:**

This study was to evaluate the predictive capability of a support vector machine (SVM) model based on serum NRG4 combined with a convolutional neural network (CNN) and long short-term memory network (LSTM)-based ultrasound feature classification (SVM-CNN+LSTM) model for predicting the occurrence of FT in patients with T2DM.

**Methods:**

Studied 500 T2DM patients (60 with FT, 440 without), and 200 healthy controls. Collected data on demographics, disease characteristics, NRG4, and thyroid indices. Pearson correlation was used to identify features correlated with NRG4. A parameter-optimized SVM model (C=1, linear kernel) was constructed for structured data modeling. Additionally, a CNN+LSTM network was employed to extract spatial (thyroid morphology) and temporal (hemodynamics) features from ultrasound sequences. These features were then fused with biochemical indicators, such as NRG4, to develop the final SVM-CNN+LSTM multimodal predictive model.

**Results:**

Serum NRG4 levels in T2DM+FT patients were significantly higher than those in the healthy Ctrl group (4.44 ± 1.25 *vs.* 2.17 ± 0.48 μg/L, *P*< 0.05). NRG4 levels were positively correlated with HOMA-IR (*r* = 0.593), FT3 (*r* = 0.773), FT4 (*r* = 0.683), thyroid volume (*r* = 0.652), and the resistance index (RI) (*r* = 0.473) (*P*< 0.05). The optimized SVM model demonstrated a sensitivity of 86.23%, specificity of 90.33%, and an area under the curve (AUC) of 0.887. In contrast, the fusion model SVM-CNN+LSTM outperformed the SVM model across all metrics, achieving a sensitivity of 91.32%, specificity of 94.18%, and an AUC of 0.943 (*P*< 0.05).

**Conclusion:**

The SVM-CNN+LSTM multimodal model, which integrates serum NRG4 levels with ultrasound features, significantly enhances the predictive accuracy of hyperthyroidism in T2DM patients. This approach effectively reveals the multifactorial mechanisms underlying T2DM-FT comorbidity, providing a powerful tool for early clinical intervention.

## 1 Introduction

Type 2 diabetes mellitus (T2DM) is a chronic metabolic disorder characterized by insulin resistance and hyperglycemia, with its global prevalence rapidly increasing. According to recent statistics, over 450 million people worldwide are affected by diabetes, the vast majority of whom have T2DM. This imposes a significant socioeconomic burden and notably increases the risk of cardiovascular diseases, renal failure, and premature mortality (1–4). In addition to glucose metabolism disorders, T2DM is frequently accompanied by various endocrine system complications, with hyperthyroidism (FT) being the most common. Epidemiological studies indicate that approximately 6–50% of T2DM patients may develop FT, further exacerbating metabolic imbalance and worsening disease prognosis (5, 6). There are multiple shared pathological mechanisms between T2DM and FT, including chronic inflammation, autoimmune dysregulation, and insulin resistance (7–9). Research suggests that excessive thyroid hormones in the FT state can promote hepatic gluconeogenesis, impair insulin sensitivity, further disrupting blood glucose control, and exacerbating diabetic complications such as neuropathy and retinopathy (10). This bidirectional interaction underscores the urgent need for early prediction of FT in T2DM patients to reduce the risk of adverse outcomes.

Neuregulin 4 (NRG4) is a novel regulatory factor secreted by adipose tissue that plays a key role in the regulation of glucose homeostasis and lipid metabolism. A substantial body of clinical and basic research has demonstrated that NRG4 enhances insulin sensitivity, inhibits hepatic gluconeogenesis, and performs various other metabolic regulatory functions. Its serum levels are negatively correlated with obesity and the severity of T2DM (11, 12). In T2DM patients, NRG4 levels are significantly reduced, suggesting its potential as a biomarker for metabolic dysfunction (13). However, there is currently no research systematically investigating the expression profile and mechanisms of NRG4 in T2DM complicated by FT. Given the high degree of overlap in metabolic pathways between T2DM and FT, NRG4 may serve as a critical hub in their interaction, warranting further investigation.

In recent years, machine learning (ML) techniques have made breakthrough progress in disease prediction, demonstrating significant potential in the management of T2DM through the integration of multimodal data to uncover complex pathological patterns. Existing studies have applied ML models to integrate clinical indicators, genetic markers, and imaging data, achieving improved risk stratification, complication prediction, and optimization of individualized treatment for T2DM (14, 15). For instance, support vector machines (SVM) and convolutional neural networks (CNN) have been used for the automated detection of diabetic retinopathy in fundus images, while random forest (RF) models have predicted cardiovascular event risks by analyzing electronic health records (16, 17). However, few studies have combined biochemical markers, such as NRG4, with dynamic imaging features for the prediction of endocrine complications in T2DM, such as FT. Traditional predictive models often rely on static clinical indicators, which struggle to capture the complex, multifactorial mechanisms between T2DM and FT, resulting in limited predictive accuracy (18).

To address the aforementioned limitations, this study proposed a multimodal machine learning-based predictive framework that integrates serum NRG4 levels with spatiotemporal features from ultrasound imaging to construct a predictive model for T2DM complicated by FT. This model combines the metabolic information of NRG4 with ultrasound morphological and hemodynamic features extracted using a CNN and long short-term memory (LSTM) network, aiming to improve prediction accuracy, enhance early diagnostic capabilities, and further elucidate the potential mechanisms of NRG4 in thyroid dysfunction and T2DM progression. This approach offers a novel strategy for early clinical intervention.

## 2 Research methodologies

### 2.1 Research design

**Figure 1:**
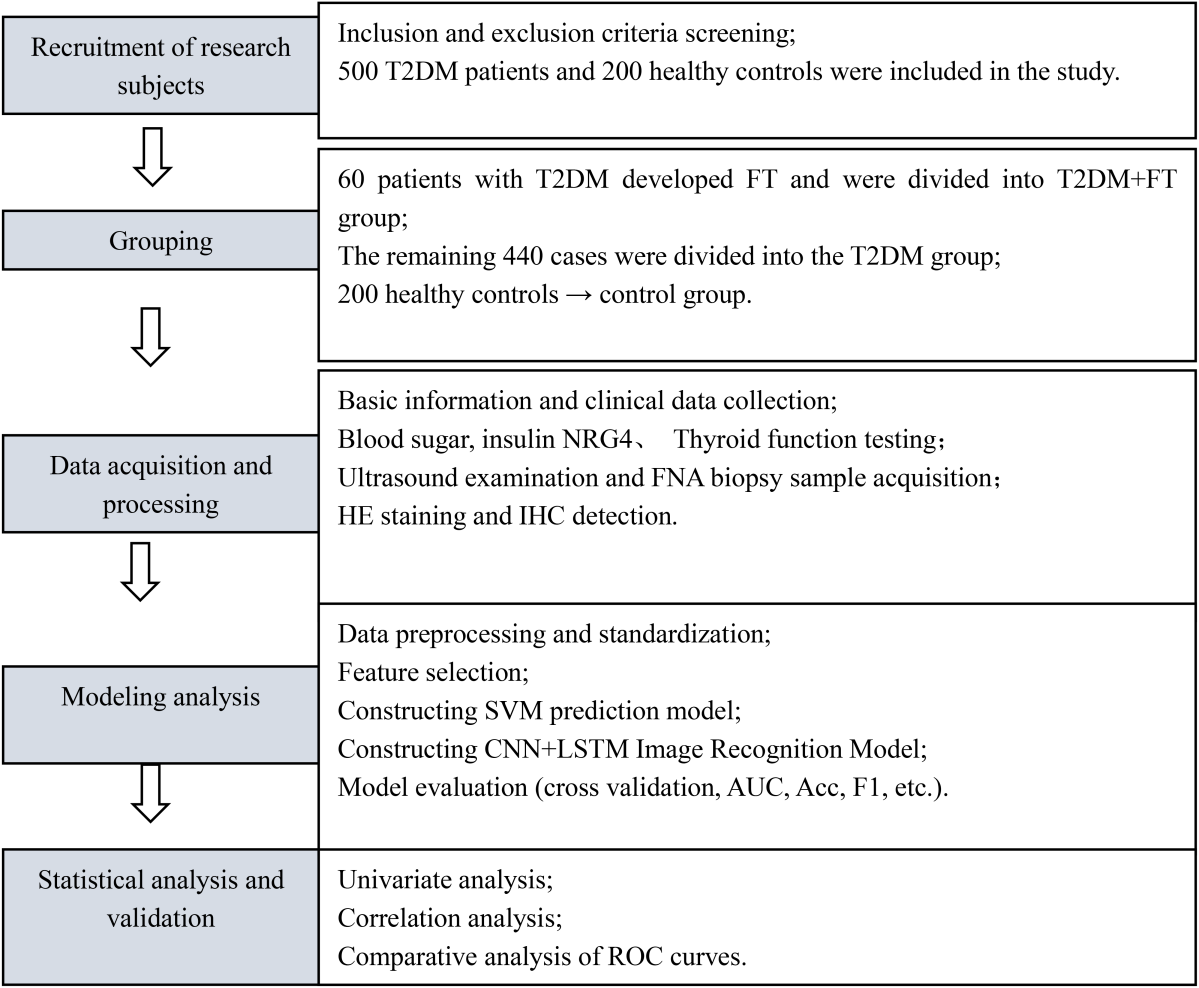
Research flowchart.

This study employed a prospective cohort design, conducting a 12-month longitudinal follow-up of all enrolled T2DM patients, with follow-up time points at baseline, 3 months, 6 months, and 12 months. Data were collected through telephone or outpatient follow-up visits, with a dropout rate of 4.2% (21/500), primarily due to patient migration or refusal to continue participation. Treatment plans for all patients (oral hypoglycemic agents or insulin injection) were standardized according to the *Chinese Guidelines for the Prevention and Treatment of Type 2 Diabetes (2017 Edition)* (19) to minimize the impact of treatment heterogeneity on the results. The detailed study flowchart is shown in **Figure 1**.

### 2.2 Research object

Patients diagnosed with T2DM admitted to the Ninth People’s Hospital of Suzhou from May 1, 2023, to April 30, 2024 were recruited, with specific inclusion and exclusion criteria as follows:

Inclusion criteria: i) diagnosis of T2DM regarding the diagnostic criteria outlined in the *Chinese Guideline for the Prevention and Treatment of Type II Diabetes Mellitus (2017 Edition)*; ii) patients with FT were diagnosed according to the diagnostic criteria outlined in the *Chinese Guideline for the Diagnosis and Treatment of Thyroid Diseases-Hyperthyroidism* (20), confirmed by thyroid color Doppler ultrasound, and excluded cases of acute or subacute thyroiditis and Hashimoto’s thyroiditis; iii) patients aged between 18 and 65 years old; iv) patients confirmed with FT had not received treatment with anti-thyroid medications prior to study participation.

Exclusion criteria: i) patients with severe cardiovascular, cerebrovascular, or vascular diseases; ii) patients with severe organ dysfunction, including liver or kidney impairment; iii) patients who received treatment for relevant diseases within the three months prior to study participation, including treatment with iodine preparations for FT; iv) patients who experienced acute metabolic disorders such as diabetic ketoacidosis within the three months prior to study participation; v) patients with malignant tumors, immune system disorders, or other endocrine disorders; vi) patients with psychiatric disorders; vii) patients with infectious diseases.

Based on the aforementioned criteria, 500 T2DM patients were included. Of which, 60 patients developed FT and were categorized into T2DM+FT group, while the remaining 440 patients did not develop FT and were categorized into T2DM group. Additionally, 200 healthy individuals undergoing routine health examinations during the same period were recruited as controls. Sample size calculation was based on previous studies (21), setting a significance level of α = 0.05 and a power of 1-β = 0.8. The expected incidence of FT in T2DM patients was 12%, and the estimated total sample size required was 480 cases. A total of 500 T2DM patients (including 60 with T2DM+FT) were actually enrolled, meeting the statistical requirements. Although the sample size in the T2DM+FT group was relatively small, the risk of overfitting was reduced through LASSO regression and cross-validation. All participants provided informed consent and agreed to participate in this work. This research adhered to the ethical standards outlined in the Helsinki Declaration and was approved by the Ethics Committee of the Ninth People’s Hospital of Suzhou (Approval Number: KY2023-029-01).

### 2.3 Data collection

Basic information (including age, gender, height, and weight), disease characteristics (including duration of diabetes), and details of diabetes treatment (including types of medication) were collected through clinical records and other data sources for all patients.

### 2.4 Blood sample collection

Fasting venous blood samples of approximately 5 mL were collected from each patient. Following blood collection, samples were centrifuged at 4,000 rpm for approximately 3 minutes using an Eppendorf 5418R centrifuge (Eppendorf AG, Germany, Catalog No: 5401000516). After centrifugation, serum portion was separated using pipettes and stored at -80°C in a Thermo Scientific Forma 900 freezer (Thermo Fisher, USA, Catalog No: 900-005) for subsequent research.

### 2.5 Blood glucose level detection

The serum samples prepared in Section 2.4 were analyzed employing an automated biochemical analyzer with corresponding reagents (Beckman Coulter AU480, Beckman Coulter, USA, Catalog No: A11857) to measure fasting blood glucose (FBG), 2-hour postprandial blood glucose (2hPBG), and hemoglobin A1c (HbA1c) levels for all patients. The normal value for FBG was defined as 5.6 mmol/L, with a diagnostic threshold for diabetes set at 7.0 mmol/L. The normal value for 2hPBG was below 7.8 mmol/L, while a value above 11.1 mmol/L was used as the criterion for diagnosing diabetes. Additionally, fasting insulin levels were measured using an automated electrochemiluminescence immunoassay (Roche Cobas e411, Roche Diagnostics, Switzerland, Catalog No: 07027742190) to calculate the homeostasis model assessment of insulin resistance (HOMA-IR).

### 2.6 Measurement of serum NRG4 levels

Serum samples collected in Section 2.4 were analyzed for serum NRG4 levels using the enzyme-linked immunosorbent assay (ELISA). The NRG4-ELISA kit was purchased from R&D Systems (USA Catalog No: EK713197), and experiments were conducted strictly regarding the manufacturer’s instructions. The concentration of NRG4 in samples was calculated using the standard curve method.

### 2.7 Thyroid function indicators

Serum samples prepared in Section 2.4 were analyzed employing automated electrochemiluminescence immunoassay to measure thyroid hormone levels, including free triiodothyronine (FT3), FT4, and thyroid-stimulating hormone (TSH) levels. The kit was Roche Diagnostics, Switzerland, Catalog No: 07027742190.

### 2.8 Thyroid tissue sampling and processing

After the informed consent was signed by the patients, a preoperative assessment was conducted to ensure that the patients were suitable for thyroid puncture. Thereafter, thyroid tissue was obtained under ultrasound guidance using fine-needle aspiration biopsy (FNA), with an adequate amount of tissue samples (1–2 mL) being collected. An ultrasound-guided procedure was performed using a GE Logiq E9 ultrasound diagnostic device (GE Healthcare, USA).

The acquired thyroid tissue samples were immediately placed in a 10% formalin fixative (Fisher Scientific, USA, catalog number: 102-097-121) for a fixation period of 24 hours. After fixation, the samples underwent dehydration and clarification processes before being embedded in paraffin to prepare tissue sections (4-5μm) for hematoxylin-eosin (H&E) staining and immunohistochemistry (IHC) examination.

### 2.9 H&E staining

Paraffin-embedded tissue sections were retrieved and deparaffinized using a graded series of hydration. Subsequently, the sections were stained with HE staining, beginning with placement in hematoxylin solution (Sigma-Aldrich, USA, catalog number: H3136) for 10 minutes, followed by rinsing with deionized water. The sections were then differentiated in hydrochloric acid alcohol (Sigma-Aldrich, USA, catalog number: 320501) for 1–3 seconds, rinsed again with deionized water, and transferred to eosin staining solution (Sigma-Aldrich, USA, catalog number: E4009) for 1–3 minutes, followed by a gentle rinse with deionized water. Finally, the sections underwent dehydration, clarification (using xylene, Sigma-Aldrich, USA, catalog number: Xylene), and mounting. The stained sections were observed under a light microscope (Olympus BX53, Japan) to record the morphology, size, and arrangement of the observed tissue cells.

### 2.10 IHC detection

The sections were baked in an oven (Thermo Scientific, USA, model: Heratherm OGS) at 60°C for one hour, deparaffinized with xylene (Sigma-Aldrich, USA, catalog number: Xylene) and ethanol (Fisher Scientific, USA, catalog number: A956-1), and then rinsed with distilled water. After being placed into 0.01 M citrate buffer (Sigma-Aldrich, USA, catalog number: C9427, pH 6.0), the sections were heated in a pressure cooker until boiling and maintained for 15 minutes. They were then quickly cooled in ice water for 5–10 minutes to terminate the reaction. After antigen retrieval, the sections were washed three times with PBS (Gibco, USA, catalog number: 10010-031) to remove unbound substances. A specific NRG4 antibody (Cell Signaling Technology, USA, catalog number: 13167S) was selected and diluted with antibody dilution solution (Beyotime, China, catalog number: P0013) at a ratio of 1:500, incubation at room temperature for one hour. The sections were then incubated with the diluted antibody in a 4°C refrigerator overnight, washed three times with PBS, and incubated with horseradish peroxidase-labeled goat anti-rabbit IgG secondary antibody (Cell Signaling Technology, USA, catalog number: 7074S) at room temperature for one hour, followed by another three washes with PBS. According to the instructions of the IHC detection kit, the DAB substrate (Zhongshan, China, catalog number: ZLI-9018) was added for the color development reaction for 5–10 minutes at room temperature. The sections were rinsed with tap water and finally mounted. The sections were observed under an optical microscope, and the expression of NRG4 was quantitatively analyzed by recording the proportion of positive cells.

### 2.11 Thyroid ultrasound examination

All patients underwent examination using a color Doppler ultrasound diagnostic device (GE Logiq E9, GE Healthcare, USA), with a high-frequency transducer (7.5–10 MHz) employed for both longitudinal and transverse scanning of the thyroid to assess its volume (calculated using the equation: Volume = Length × Width × Height × 0.52). Blood flow in the thyroid was evaluated, with blood flow velocity and resistance index (RI) recorded for various parts, and the blood supply to the thyroid was analyzed.

### 2.12 Machine learning algorithms

#### 2.12.1 Model structure

(1) Data collection and preprocessing: clinical data, serum NRG4 levels, thyroid function-related indicators (such as TSH, T3, T4), and imaging data of T2DM patients were collected. The data were cleaned, and missing or outlier values were addressed. Standardization (Z-score normalization) was applied to the features to ensure that all features have the same dimensional scale.

(2) Feature selection: Pearson correlation coefficient was first used to calculate the linear or nonlinear relationships between features and the target variable (FT) to identify features related to the target variable. Subsequently, LASSO regression was applied for feature selection to prevent overfitting and improve the model’s generalization ability.

(3) SVM prediction model: In this study, SVM was used to classify patients into two groups: “Diabetes with FT” and “Diabetes without FT”. Given the training set {(χ_i_),}, where χ_i_∈R_i_ represents the input features, and γ∈{−1,+1} represents the class label. The objective function is given in **Equation 1**


(1) $$\begin{array}{c} min\\ \beta ,b \end{array}\frac{1}{2}\|\beta^{2}\|\;subject\;to\;y_{i}\left((\beta ,x_{i})\right)+b\geq 1\;\forall i$$

Where β is the normal vector of the hyperplane, *b* is the bias term, and <β>+=0 represents the equation of the hyperplane. Subsequently, the study selected an appropriate kernel function to enhance the classification ability of the SVM model and adapt to different data distributions.

(4) Ultrasound image feature extraction based on deep learning: a CNN combined with a long short-term memory network (LSTM) was employed for automatic feature extraction from ultrasound images. In this approach, CNN is used to extract local spatial features from the ultrasound image data, which primarily includes convolutional layers (Conv2D), pooling layers (MaxPooling2D), batch normalization (BatchNorm), and fully connected layers (Dense), among others. The input shape is 224×224×3 (*i.e.*, color ultrasound images with a resolution of 224×224), and standard preprocessing is applied to normalize the pixel values within the range of 0 to 1 to enhance model stability. The convolutional layers (Conv2D) utilize multiple 3×3 filters for feature extraction, with the activation function being the ReLU non-linear activation function. The pooling layers apply 2×2 max pooling (MaxPooling) to reduce the image dimensions, decreasing computational complexity while retaining key features. The output of the convolutional layers is then batch-normalized to accelerate convergence and improve training stability. The Flatten layer converts the final feature map into a one-dimensional vector, preparing it for input into the LSTM network.

LSTM networks are a specialized type of recurrent neural network (RNN) capable of learning temporal sequence features from ultrasound images. In this study, LSTM was used to process continuous ultrasound frame sequences, capturing the disease progression trend and enhancing the temporal consistency of predictions. The LSTM network structure takes as input data with the dimensions (batch_size, time_steps, features), where “batch_size” represents the number of samples, “time_steps” refers to the number of frames in the sequence, and “features” corresponds to the number of features extracted by the CNN. The time step is set to 5–10 frames, meaning each input group contains 5–10 consecutive ultrasound images. The LSTM unit’s computational flow involves the Forget Gate, Input Gate, Cell State, and Output Gate, where the gating mechanism enables selective information transmission and memory retention. The feature vector processed by the LSTM is then passed through a fully connected layer (Dense) and subjected to binary classification using a Softmax activation function.

#### 2.12.2 Training and optimization

The performance of SVM prediction models under different *C* parameters and kernel functions was evaluated using accuracy, *Recall*, and *F1* score metrics. Hyperparameter tuning was primarily conducted through cross-validation to obtain the optimal model performance.

For each hyperparameter combination, *k*-fold cross-validation was employed. The 350 examples in the training set were divided into 50 subsets, with each iteration using 50−1 subsets for training and one subset for validation. This process was repeated 50 times for cross-validation, and the average was taken as the evaluation result. The calculation methods for accuracy, Recall, and F1-score are detailed in **Equation 2**–**4**:


(2) $$Acc=\frac{TP+TN}{TP+FP+FN+TN}$$

(3) Recall=TPTP+FN

(4) F1=2×(TPTP+FP)×Recall(TPTP+FP)+Recall

*TP* represents correctly predicted positive instances, *FP* represents incorrectly predicted positive instances, *FN* represents incorrectly predicted negative instances, and *TN* represents correctly predicted negative instances.

Classification performance evaluation of CNN+LSTM: to assess the performance of the CNN+LSTM model in classification tasks, this study employed an ablation study to validate the model’s effectiveness. The model structure ablation is as follows:

CNN model: the LSTM layer is removed, using only the CNN for feature extraction and classification.

LSTM model: the CNN layer is removed, and only LSTM is used to process the time-series data.

CNN+LSTM model: the complete CNN+LSTM model, combining the spatial feature extraction capability of CNN with the temporal sequence modeling ability of LSTM.

The evaluation metrics include accuracy, recall, and F1 score, with training and validation performed similarly to the SVM model.

### 2.13 Statistical analysis

Statistical analysis was performed using *SPSS 22.0* (IBM Corporation, USA). Categorical variables (expressed as percentages) were compared using the chi-square test (χ^2^ test). For continuous variables that followed a normal distribution, one-way analysis of variance (ANOVA) was used, and pairwise comparisons between groups were conducted using Tukey’s *post hoc* test. To account for the risk of Type I errors due to multiple comparisons, the Bonferroni correction was applied to adjust the significance level to α = 0.0045 (0.05/11 indicators). Pearson correlation analysis was employed to assess the association between serum NRG4 levels and clinical parameters (*e.g.*, HOMA-IR, FT3, TSH), with the Holm-Bonferroni method used to adjust the significance threshold for multiple correlation analyses. The predictive performance of the SVM model and traditional NRG4 levels was evaluated through receiver operating characteristic (ROC) curve analysis, and the area under the curve (AUC) was calculated to quantify overall accuracy. Unless otherwise specified for threshold adjustments, a two-tailed test with *P*< 0.05 was considered statistically significant.

## 3 Results

### 3.1 Comparison of baseline data among three groups of patients

The baseline data were collected from Ctrl group, T2DM group, and T2DM+FT group, including age, gender, height, weight, duration of T2DM, and type of T2DM treatment medications (**Table 1**). Upon comparison, no considerable differences were observed in the baseline characteristics among Ctrl group, T2DM group, and T2DM+FT group (*P*>0.05).

**Table 1:** Baseline data of three groups of patients.

Group		Ctrl group (n=200)	T2DM group (n=440)	T2DM+FT group (n=60)	*P*
Age (years old)		54.12 ± 6.39	57.02 ± 6.11	55.29 ± 7.33	0.283
Sex n (%)	Female	110 (45)	268 (60.91)	35 (58.33)	0.313
	Male	90 (55)	172 (39.09)	25 (41.67)	0.093
Height (cm)		170.09 ± 10.16	167.21 ± 11.23	173.02 ± 9.24	0.082
Weight (kg)		59.37 ± 5.33	61.34 ± 6.03	56.93 ± 5.39	0.056
T2DMdrug n (%)	Oral class	——	251 (57.05)	30 (50)	0.088
	Injection	——	145 (32.95)	23 (38.33)	0.183
	Others	——	44 (10)	7 (11.67)	0.280
T2DM disease course (years)		——	4.37 ± 1.29	4.46 ± 1.22	0.111

### 3.2 Comparison of blood glucose, NRG4 levels, and thyroid indices

**Table 2** summarizes the measurement results of FBG, 2hPBG, HbA1c, HOMA-IR, NRG4, FT3, FT4, and TSH levels in patients of the Ctrl group, T2DM group, and T2DM+FT group. The results showed that the levels of FBG, 2hPBG, HbA1c, and NRG4 in the T2DM and T2DM+FT groups were significantly higher than those in the Ctrl group (*P*<0.05), and the level of HOMA-IR in the T2DM+FT group was also significantly increased (*P*<0.05). Furthermore, the T2DM+FT group exhibited significantly higher levels of FT3 and FT4, as well as significantly lower levels of TSH (*P*<0.05).

**Table 2:** Comparison of clinical and laboratory indicators among groups.

Indicators	Ctrl group (n=200)	T2DM group (n=440)	T2DM+FT group (n=60)
FBG (mmol/L)	4.69 ± 0.39	8.80 ± 0.84**	9.36 ± 2.34**
2hPBG (mmol/L)	6.16 ± 0.37	16.73 ± 0.42**	16.43 ± 1.42**
HbA1c (%)	5.18 ± 0.44	8.56 ± 1.01**	8.12 ± 1.12**
HOMA-IR	2.30 ± 0.27	2.48 ± 0.53	5.27 ± 0.89**^
NRG4 (μg/L)	2.17 ± 0.48	3.53 ± 1.22**	4.44 ± 1.25**^
FT3 (pmol/L)	3.40 ± 0.48	3.58 ± 0.42**	17.29 ± 1.28**^
FT4 (pmol/L)	10.11 ± 2.17	11.29 ± 1.83**	40.32 ± 2.90**^
TSH (mU/L)	2.60 ± 0.46	2.69 ± 0.57	0.21 ± 0.04**^

**compared with the Ctrl group, *P*<0.05; ^compared with the T2DM group, *P*<0.05.

### 3.3 Histopathological analysis

#### 3.3.1 Analysis of H&E staining results

**Figure 2:**
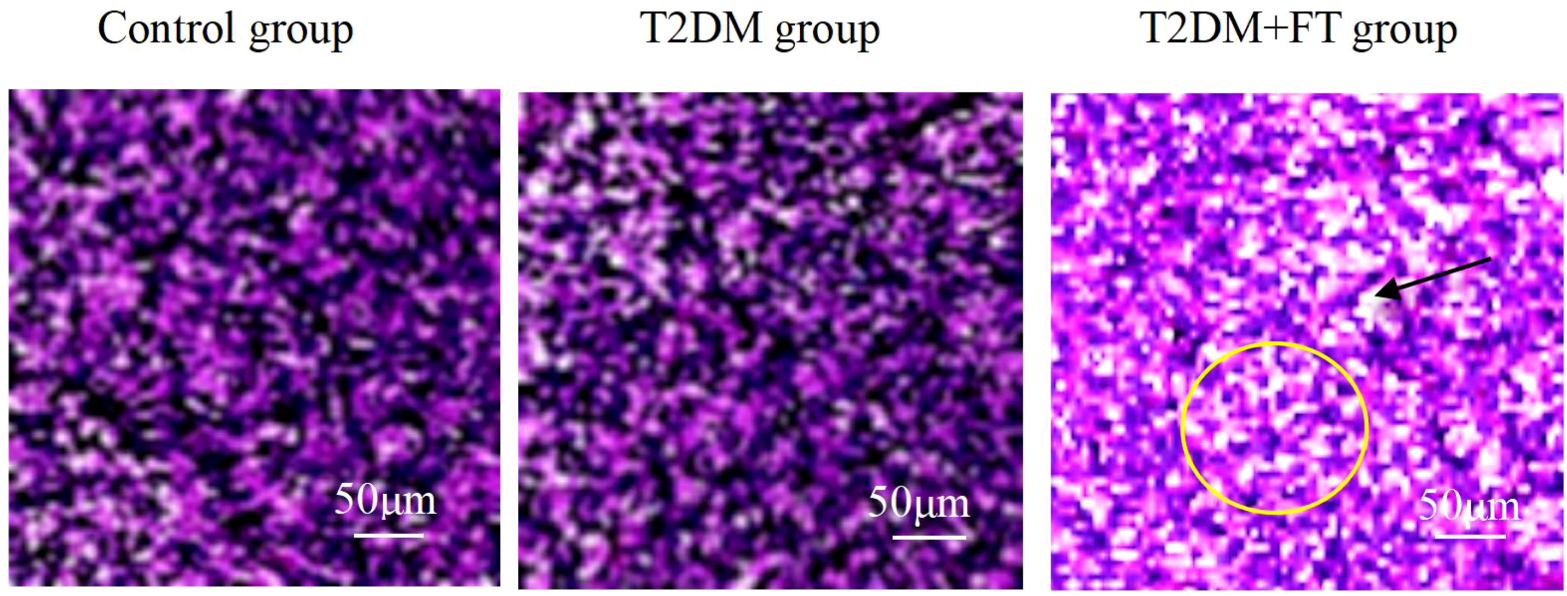
Comparison of H&E staining results among the three groups.

In thyroid tissue samples, H&E staining results showed that in the T2DM+FT group, thyroid cells exhibited significant proliferation with irregular cell arrangement (indicated by yellow circles), and there was marked interstitial edema (indicated by black arrows). Compared to the T2DM group, the T2DM+FT group showed enlarged cell nuclei, expanded cytoplasm, and the presence of interstitial fluid accumulation. The H&E staining results for the T2DM group indicated a more regular cell arrangement, mild degree of proliferation, and no significant interstitial edema. The thyroid tissue of the healthy Ctrl group displayed normal cellular morphology and structure, with tightly packed cell arrangement and very little interstitial fluid accumulation (**Figure 2**).

#### 3.3.2 IHC results

**Figure 3:**
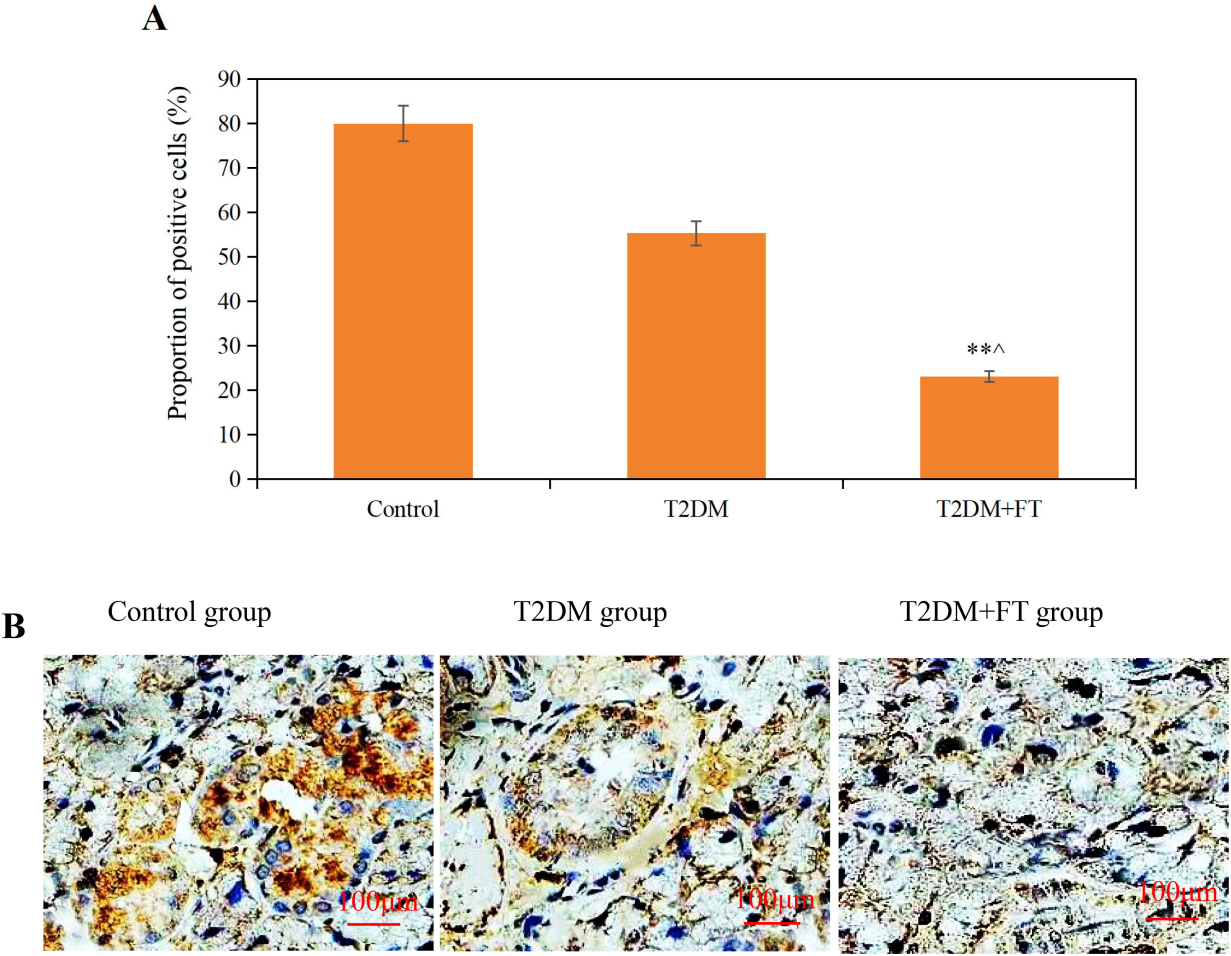
Comparative analysis of IHC results among the three groups [**(A)** Proportion of NRG4-positive cells **(B)** IHC images; ** compared with the Ctrl group, *P*<0.05; ^ compared with the T2DM group, *P*<0.05].

The IHC results indicated that the expression of NRG4 in thyroid tissue was significantly reduced in the T2DM+FT group (*P*<0.01), with a markedly lower proportion of positive cells compared to the T2DM group and the Ctrl group. The proportion of NRG4-positive cells in the T2DM+FT group was only 23.09 ± 1.22%, while in the T2DM group it was 55.33 ± 3.32%, and in the Ctrl group it was as high as 80.02 ± 4.02% (**Figure 3**). IHC images clearly demonstrate the differences in NRG4 expression between the groups. The brownish areas in the T2DM+FT group were significantly lower compared to the control and T2DM groups, further supporting the role of NRG4 as a potential biomarker.

### 3.4 Comparative analysis of ultrasound detection results

**Figure 4:**
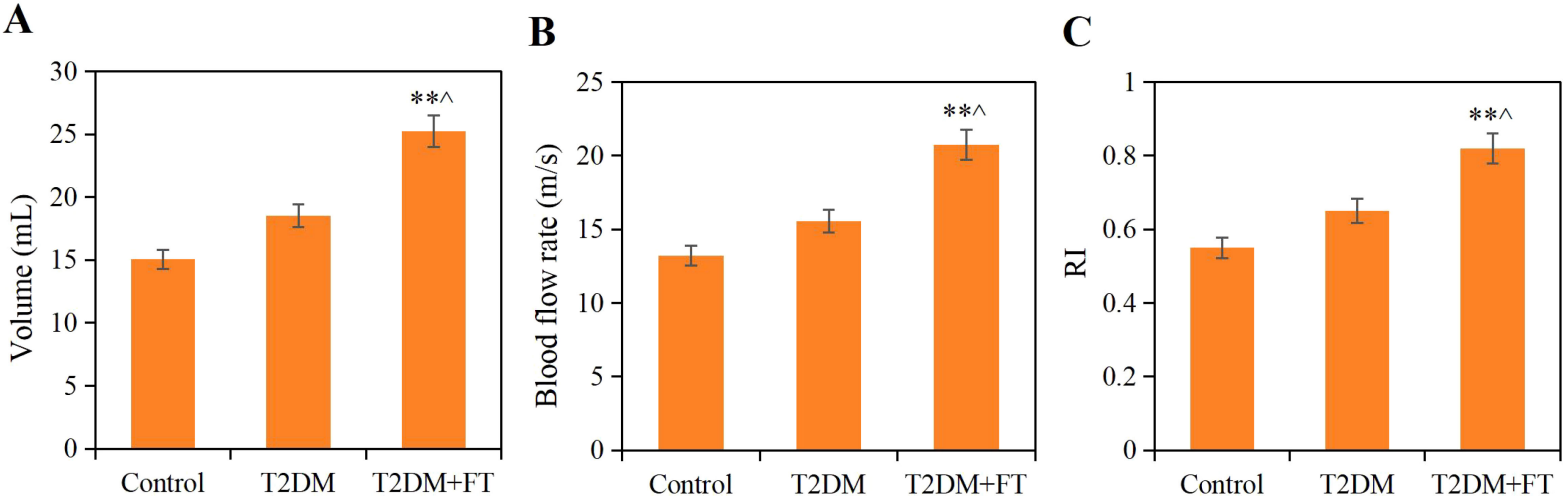
Comparison of ultrasound detection results among the three groups **(A)** Thyroid volume **(B)** Blood flow velocity **(C)** RI; ** compared with the Ctrl group, *P*<0.05; ^ compared with the T2DM group, *P*<0.05).

All patients underwent neck ultrasound examinations to assess the structure and blood flow of the thyroid gland. The results showed that the volume of the thyroid gland in patients of the T2DM+FT group was significantly enlarged to (25.24± 6.14) mL, which was significantly different compared to the T2DM group (18.52 ± 4.33) mL and the Ctrl group (15.06 ± 3.8 mL) (*P*<0.05). Additionally, the blood flow velocity in the T2DM+FT group (20.75 ± 3.52) cm/s was also significantly higher than that in the T2DM group (15.55 ± 2.29) cm/s and the Ctrl group (13.21 ± 2.09) cm/s, while the RI in the T2DM+FT group (0.82 ± 0.08) was significantly higher than that in the T2DM group (0.65 ± 0.05) and the Ctrl group (0.55 ± 0.06) (*P*<0.05) (**Figure 4**).

### 3.5 Correlation of serum NRG4 with related indicators

Based on the statistical results from Sections 3.2 and 3.3, Pearson’s correlation coefficient was employed to assess the relationship between NRG4 levels in patients with T2DM combined with FT and various indicators, including FBG, 2hPBG, HbA1c, HOMA-IR, FT3, FT4, TSH, the positive rate of NRG4 in thyroid tissue, thyroid volume, blood flow velocity, and RI (**Table 3**). The levels of NRG4 were found to have a significant positive correlation with HOMA-IR, FT3, FT4, thyroid volume, blood flow velocity, and RI, and a significant negative correlation with the positive rate of NRG4 in thyroid tissue and TSH (*P*<0.05).

**Table 3:** Correlation of NRG4 with FBG, 2hPBG, HbA1c, HOMA-IR, FT3, FT4, and TSH in T2DM patients with concurrent FT.

Correlation	NRG4	
	r	P
FBG	0.183	0.403
2hPBG	0.128	0.684
HbA1c	-0.011	1.212
HOMA-IR	0.593	0.008
FT3	0.773	0.000
FT4	0.683	0.000
TSH	-0.809	0.000
Positive rate of NRG4 in thyroid tissue	-0.122	0.009
Thyroid volume	0.652	0.002
Blood flow velocity	0.589	0.013
RI	0.473	0.010

### 3.6 SVM parameter optimization

**Figure 5:**
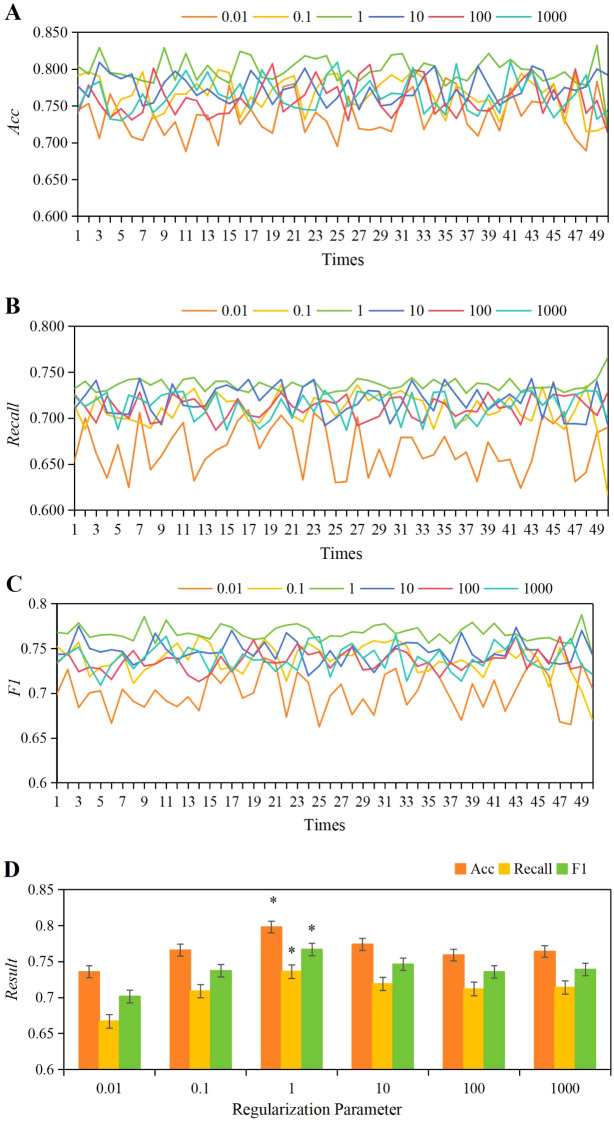
The variations in accuracy **(A)**, *Recall* **(B)**, and *F1* score **(C)** under different *C* parameters, along with a comparison of their average values **(D)**. *P<0.05 vs. *C* parameters of 0.01, 0.1, 10, 100, and 1,000.

Through *k*-fold cross-validation analysis, different values of *C* parameters and kernel functions were evaluated based on accuracy, *Recall*, and *F1* score values to determine the optimal parameters. The specific results are as follows:

For *C* parameters of 0.01, 0.1, 1, 10, 100, and 1,000, the 50 iterations of training yielded accuracy, *Recall*, and *F1* score values as shown in **Figures 5A–C**. Observing the results, it was found that when *C*=1, the fluctuations in accuracy, *Recall*, and *F1* scores across the 50 training iterations were the most stable. As depicted in **Figure 5D**, the average values for accuracy, *Recall*, and *F1* scores were as follows: *C* = 0.01: accuracy = 0.736 ± 0.028, *Recall* = 0.667 ± 0.026, *F1 =* 0.701 ± 0.021; *C* = 0.1: accuracy = 0.766 ± 0.025, *Recall* = 0.709 ± 0.020, *F1 =* 0.738 ± 0.018; *C* = 1: accuracy = 0.798 ± 0.019, *Recall* = 0.736 ± 0.007, *F1 =* 0.767 ± 0.009; *C* = 10: accuracy = 0.774 ± 0.010, *Recall* = 0.719 ± 0.017, *F1 =* 0.747 ± 0.013; *C* = 100: accuracy = 0.759 ± 0.024, *Recall* = 0.712 ± 0.012, *F1 =* 0.736 ± 0.012; *C* = 1,000: accuracy = 0.764 ± 0.023, *Recall* = 0.714 ± 0.015, *F1 =* 0.739 ± 0.014. By comparison, when *C* = 1, the average values of accuracy, *Recall*, and *F1* score were significantly higher than those at *C* = 0.01, 0.1, 10, 100, and 1,000 (*P*<0.05).

**Figure 6:**
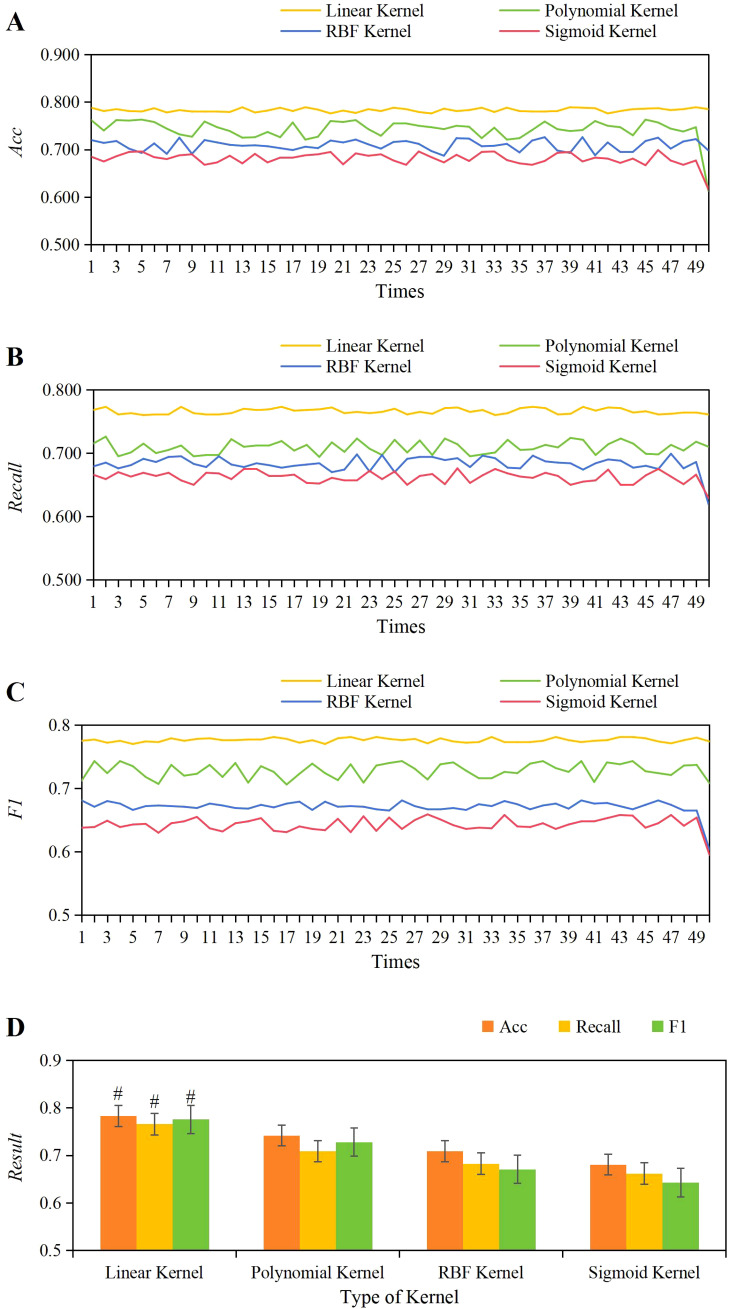
The variations in accuracy **(A)**, *Recall* **(B)**, and *F1* score **(C)** across different kernel functions, along with a comparison of their average values **(D)**. #*P*<0.05 vs. the polynomial, RBF, and Sigmoid kernel functions.

Based on the results obtained, the optimal value for the *C* parameter was determined to be 1, guiding the determination of the optimal kernel function for this work. The evaluation compared the performance metrics (accuracy, *Recall*, *F1*) across various kernel functions including linear, polynomial, RBF, and Sigmoid, over 50 training iterations as shown in **Figures 6A–C**. Observations revealed that the variations in accuracy, *Recall*, and *F1* scores were most stable across 50 training iterations using the linear kernel function. As depicted in **Figure 6D**, the average values for the linear kernel were accuracy = 0.783 ± 0.004, *Recall* = 0.766 ± 0.004, *F1 =* 0.776 ± 0.003; for the polynomial kernel, accuracy = 0.742 ± 0.023, *Recall* = 0.709 ± 0.010, *F1 =* 0.728 ± 0.012; for the RBF kernel, accuracy = 0.709 ± 0.011, *Recall* = 0.683 ± 0.012, *F1 =* 0.671 ± 0.011; and for the Sigmoid kernel, accuracy = 0.681 ± 0.03, *Recall* = 0.662 ± 0.009, *F1 =* 0.643 ± 0.011. Comparison analysis revealed that the average accuracy, *Recall*, and *F1* values of the linear kernel were greatly higher than those of the polynomial, RBF, and Sigmoid kernel functions (*P*<0.05).

### 3.7 Ablation study results of the CNN+LSTM model

**Figure 7:**
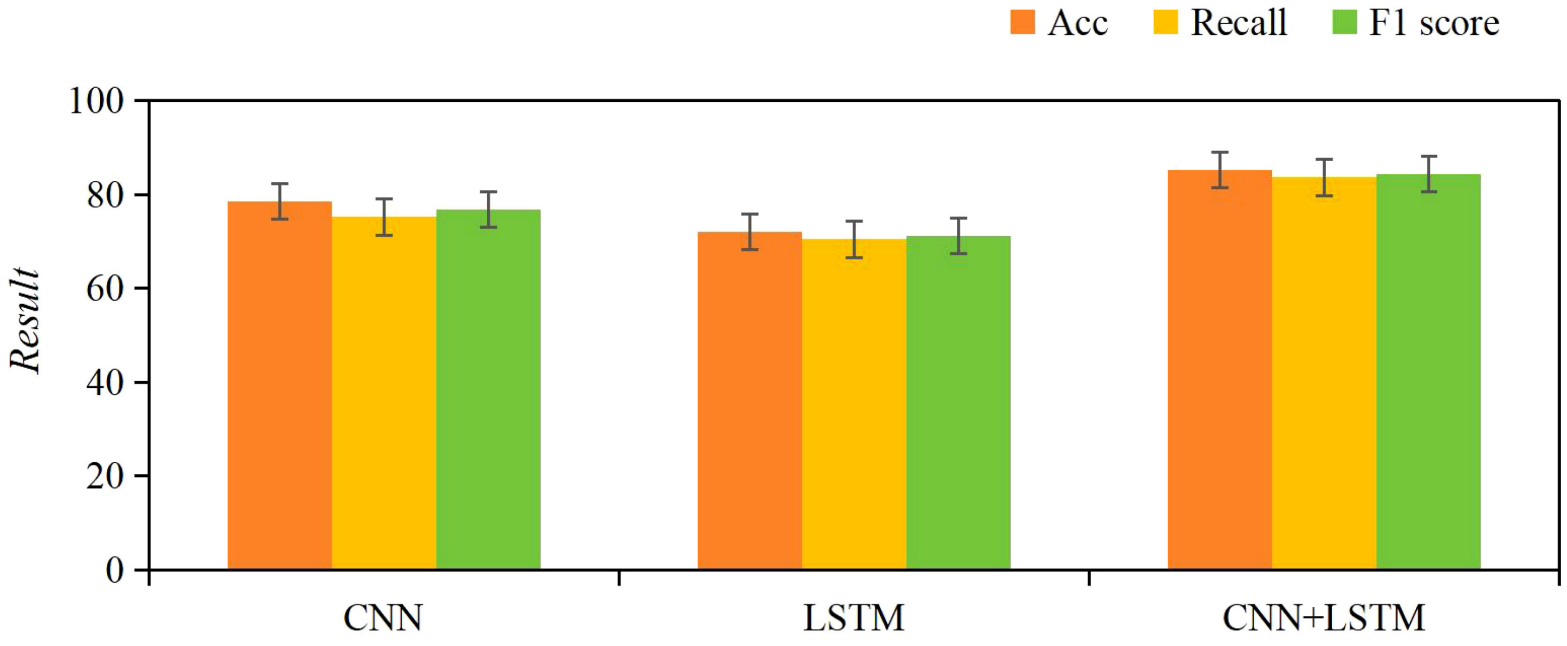
Comparison of classification performance between CNN, LSTM, and CNN+LSTM models.

The results of the ablation study showed that the CNN model achieved an accuracy of 78.5%, recall of 75.3%, and F1 score of 76.8%, while the LSTM model achieved an accuracy of 72.1%, recall of 70.5%, and F1 score of 71.3%. In contrast, the CNN+LSTM model significantly outperformed both individual models, with an accuracy of 85.2%, recall of 83.7%, and F1 score of 84.4%. These results demonstrate that the combination of CNN and LSTM better captures the spatial features and temporal sequence characteristics of ultrasound images (**Figure 7**).

### 3.8 Prediction performance of the SVM model based on NRG4 levels combined with ultrasound feature classification based on CNN+LSTM

**Figure 8:**
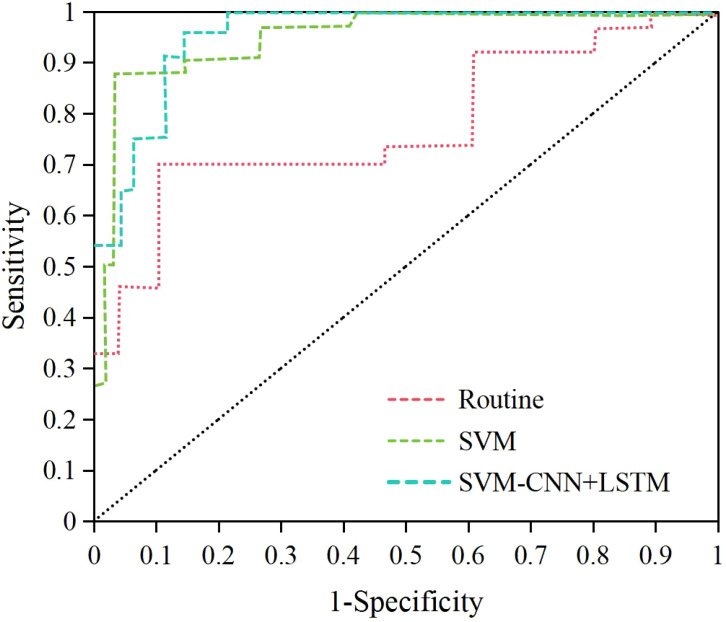
ROC curves of different methods and models for predicting T2DM complicated by FT.

Based on the above, it was known that NRG4 showed a significant correlation with thyroid indicators such as FT3, FT4, TSH, HOMA-IR, the positive rate of NRG4 in thyroid tissue, thyroid volume, blood flow velocity, and RI. Therefore, this study obtained the NRG4 levels of patients with isolated T2DM and those with T2DM complicated by FT. An SVM model based on NRG4 levels combined with an ultrasound feature classification prediction model based on CNN+LSTM (SVM-CNN+LSTM) was applied to evaluate its predictive performance for T2DM complicated by FT and compared with traditional NRG4-based prediction methods. The ROC curve was plotted, and the results are shown in **Figure 8**. The area under the ROC curve (AUC = 0.943) for the SVM-CNN+LSTM model was significantly higher than that of the traditional method (AUC = 0.732). The optimal cutoff value for this model corresponded to a sensitivity of 91.32% and a specificity of 94.18%.

**Figure 9:**
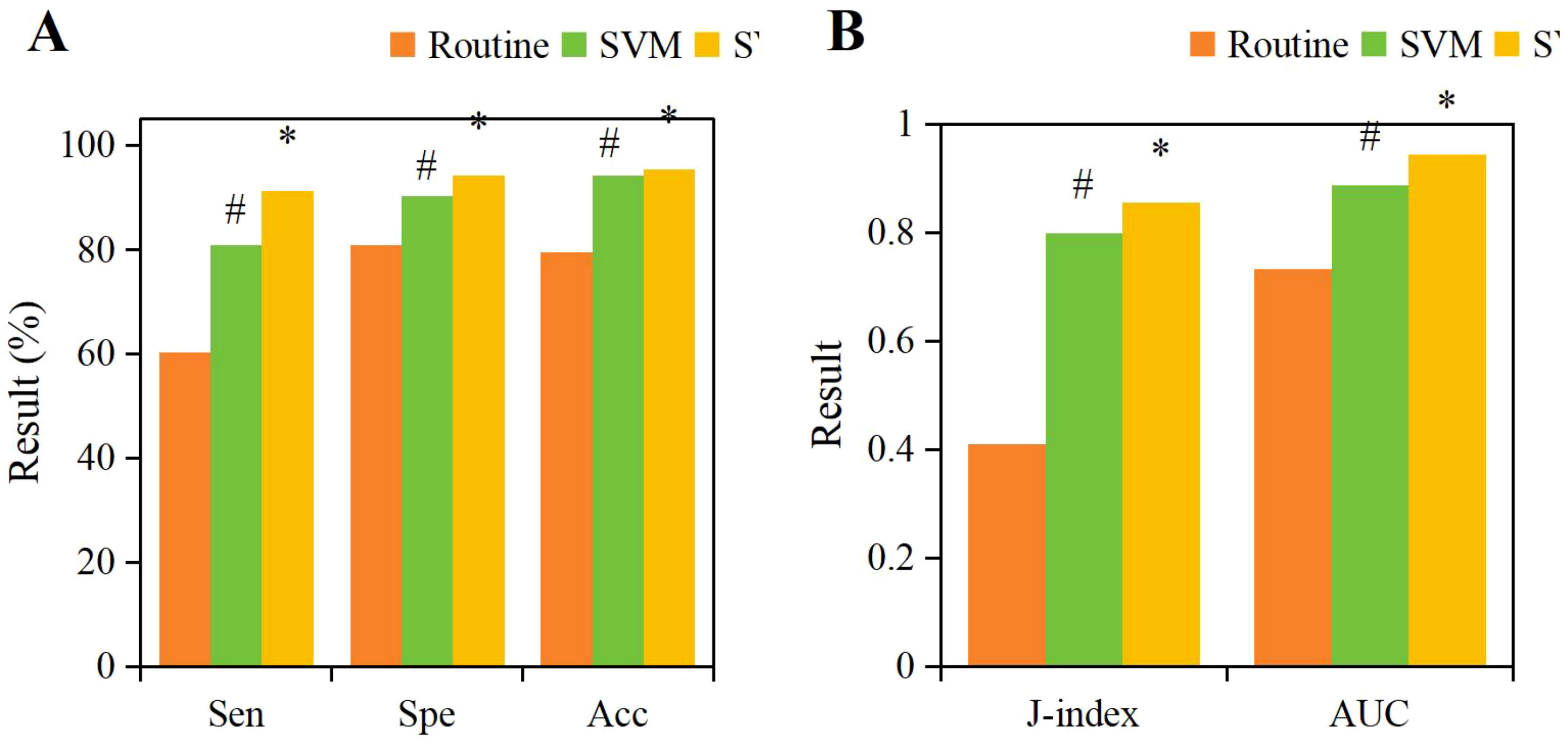
Comparison of specificity **(A)**, specificity **(A)**, accuracy **(A)**, Youden index **(B)**, and AUC **(B)** for different prediction methods. (#*P*<0.05, compared to the traditional NRG4 prediction method, *P<0.05, compared to the SVM prediction model.).

According to the ROC curve, the specificity, specificity, accuracy, Youden index, and AUC of the SVM-CNN+LSTM classification model for prediction were 91.32%, 94.18%, 95.35%, 0.855, and 0.943, respectively. The prediction results of the SVM model based on NRG4 levels were 86.23%, 90.33%, 90.89%, 0.798, and 0.887, respectively. In comparison, the traditional NRG4 prediction method yielded specificity, specificity, Youden index, and AUC of 60.22%, 80.78%, 79.49%, 0.410, and 0.732 (**Figure 9**). The SVM model based on NRG4 levels showed significant improvement over the traditional prediction method in terms of specificity, specificity, Youden index, and AUC, while the SVM-CNN+LSTM classification model further outperformed the NRG4-based SVM model in all prediction metrics (*P*<0.05).

## 4 Discussion

The rising global prevalence of T2DM and its frequent comorbidity with FT (incidence rate of 7%-50%) (22, 23) underscores the necessity for early prediction tools. Our findings indicate that, compared to T2DM alone, T2DM+FT patients exhibit significantly higher levels of HOMA-IR and NRG4 (*P*< 0.05), suggesting an exacerbation of insulin resistance and metabolic dysfunction. These interactions may accelerate disease progression (24), highlighting the need for targeted interventions.

Research has shown that NRG4 plays a role in regulating lipid metabolism and insulin sensitivity (25–27). In the current study, the decreased expression of NRG4 in thyroid tissue of T2DM+FT patients (*P*< 0.01) may lead to abnormal cell proliferation. In this cohort, elevated serum NRG4 levels were positively correlated with HOMA-IR, FT3, and FT4 (*P*< 0.05) and negatively correlated with TSH (*P*< 0.05), indicating a compensatory response to thyroid hormone dysregulation. Mechanistically, thyroid hormones may upregulate NRG4 through the PI3K/Akt signaling pathway (28), consistent with preclinical models (29, 30). However, due to unmeasured confounding factors, these associations must be interpreted with caution. For instance, metformin (a common antidiabetic medication) regulates thyroid function (31), and lifestyle factors such as iodine intake may alter thyroid hemodynamics (32). Future studies should employ propensity score matching to isolate drug effects and adjust for lifestyle covariates.

Additionally, pathological and imaging analyses revealed that thyroid tissue samples from patients with T2DM complicated by FT demonstrated significant cellular hyperplasia, irregular cell arrangement, and marked interstitial edema. Furthermore, IHC results further confirmed the significant reduction of NRG4 in thyroid tissue in the T2DM+FT group. NRG4 plays an important role in the proliferation and differentiation of thyroid cells, and its decreased expression may lead to abnormal proliferation of thyroid cells, thereby affecting the normal function of the thyroid gland (33, 34). Specifically, the expression of NRG4 is closely related to the proliferation and differentiation of thyroid cells, and its decline may lead to abnormal proliferation of thyroid cells. Ultrasound examination results indicated that patients in the T2DM+FT group had significantly abnormal thyroid volume, blood flow velocity, and RI, further reflecting the changes in thyroid hemodynamics. These changes may be due to increased metabolic activity caused by FT, which in turn affects the blood supply and structure of the thyroid gland (35). Correlation analysis also confirmed that serum NRG4 was significantly positively correlated with thyroid volume, blood flow velocity, and RI, and significantly negatively correlated with the positive rate of NRG4 in thyroid tissue.

To address the limitations of traditional prediction models that rely solely on clinical indicators and biochemical markers (36), this study developed a multimodal prediction model based on SVM and SVM-CNN+LSTM, which significantly improved predictive performance. The results show that the NRG4-based SVM model outperformed traditional methods in sensitivity (86.23%), specificity (90.33%), and AUC (0.887) (*P*< 0.05). Relevant studies indicate that SVM has unique advantages in handling high-dimensional data and nonlinear relationships, and has been widely applied in the prediction and diagnosis of cancer, cardiovascular diseases, and diabetes (37–41). Further integration of spatiotemporal features extracted by CNN+LSTM (such as thyroid volume, blood flow velocity, and RI) with NRG4 levels led to a multimodal model with sensitivity, specificity, AUC, and Youden index of 91.32%, 94.18%, 0.943, and 0.855, respectively, all significantly higher than those of the single SVM model (*P*< 0.05). This multi-level feature fusion strategy not only enhanced the model’s classification performance but also provided new insights into the pathological interactions between T2DM and FT (42, 43). Despite the excellent predictive performance of the model, its practical application still requires standardized operational procedures. It is recommended to simultaneously collect imaging and serum samples during routine ultrasound examinations, standardize the extraction of NRG4 and ultrasound parameters, and establish an auxiliary decision-making system for real-time risk assessment. Additionally, an operational manual should be developed and training programs implemented to ensure standardized use of the model. In the future, integrating with clinical information systems will further validate and promote its application value in routine screening.

Furthermore, this study validated the feature representation capability of the multimodal model using t-SNE visualization. The T2DM+FT group and the Ctrl group formed distinct clusters in the low-dimensional space, demonstrating that the model effectively distinguishes between disease states. When combined with pathological findings, such as the reduced expression of NRG4 in thyroid tissue and abnormal cellular proliferation, as well as ultrasound parameters, including increased thyroid volume and hemodynamic changes, the results further support the biological association between NRG4 and thyroid dysfunction. These findings also highlight the potential for clinical translation of the multimodal model. Other studies also demonstrated that machine learning-based multimodal strategies can significantly enhance the robustness of disease prediction. For instance, SVM combined with radiomics features has shown excellent performance in cancer risk stratification (44), while the CNN-LSTM model has exhibited advantages in time-series modeling for cardiovascular event prediction (45). The results of this study align with such cutting-edge work, confirming the vast potential of multimodal artificial intelligence models in the prediction of metabolic-endocrine diseases.

Although our model demonstrates high accuracy, its generalizability may be limited by the single-center design and potential confounding factors such as medication heterogeneity. Therefore, future studies will involve multi-center, large-sample prospective cohort research, with the T2DM+FT group reaching at least 100 cases. Sample collection and follow-up validation are planned to be completed within the next 2–3 years. Additionally, the molecular mechanisms of NRG4 in T2DM with FT will be further explored, such as by integrating genomics and metabolomics data, focusing on the regulatory role of signaling pathways like PI3K/Akt. Consideration may also be given to incorporating attention mechanisms to optimize feature fusion algorithms. A detailed experimental design and inter-institutional collaboration plan will be developed, with clear goals and timelines for each phase to ensure data standardization and result reproducibility.

## 5 Conclusion

This study confirmed the value of NRG4 in predicting T2DM with FT by analyzing its correlation with multiple clinical indicators. The SVM-based predictive model improved the predictive efficacy of NRG4, highlighting its potential as an independent biomarker. The multimodal model, constructed by combining spatiotemporal features extracted by CNN+LSTM, further enhanced predictive performance, demonstrating the advantages of multimodal data integration in analyzing the interactions between T2DM and FT. The findings provide new insights for the early identification and mechanistic research of T2DM with FT.

## Data Availability

The original contributions presented in the study are included in the article/supplementary material. Further inquiries can be directed to the corresponding author.
